# Analysis of tirzepatide in the US FDA adverse event reporting system (FAERS): a focus on overall patient population and sex-specific subgroups

**DOI:** 10.3389/fphar.2024.1463657

**Published:** 2024-11-06

**Authors:** Yingyong Ou, Zhiwei Cui, Siyu Lou, Chengyu Zhu, Junyou Chen, Linmei Zhou, Ruizhen Zhao, Li Wang, Fan Zou

**Affiliations:** ^1^ Department of Respiratory and Critical Care Medicine, Affiliated Hospital of Zunyi Medical University, Zunyi, China; ^2^ Department of Obstetrics and Gynecology, The First Affiliated Hospital of Xi’an Jiaotong University, Xi’an, China

**Keywords:** tirzepatide, FAERS, adverse drug events, disproportionality analysis, real-world analysis

## Abstract

**Objective:**

Tirzepatide, a novel GIP and GLP1 agonist, has been extensively examined in clinical trials. However, specific data on its adverse drug events (ADEs) remain limited. This study aims to comprehensively assess real-world ADEs associated with tirzepatide by mining data from the U.S. Food and Drug Administration Adverse Event Reporting System (FAERS) database.

**Methods:**

ADE reports from the FAERS database were retrieved for the second quarter of 2022 through the first quarter of 2024. Significant associations between ADEs and tirzepatide were evaluated using proportional disproportionality analyses, including the Reporting Odds Ratio (ROR), Proportional Reporting Ratio (PRR), Bayesian Confidence Propagation Neural Network (BCPNN), and Multi-item Gamma Poisson Shrinkage (MGPS).

**Results:**

A total of 37,827 ADE reports associated with tirzepatide were identified, with 100 significantly disproportionate preferred terms (PTs) recognized by all four algorithms. The top five PTs with the highest reporting rates were incorrect dose administered, injection site pain, off-label use, nausea, and injection site hemorrhage. Additionally, unexpected signals such as starvation ketoacidosis were identified. The median time to onset for all ADEs was 23 days. Furthermore, sex-specific high-intensity signals were found, with males primarily experiencing gastrointestinal disorders and females experiencing general disorders and administration site conditions.

**Conclusion:**

This study provides valuable insights into the occurrence of ADEs following tirzepatide administration, potentially supporting clinical monitoring and risk identification efforts.

## Background

Tirzepatide is a novel glucose-dependent insulinotropic polypeptide (GIP) and glucagon-like peptide-1 (GLP-1) receptor dual agonist (Frias et al., 2018). This dual-pathway intervention enhances efficacy through synergistic responses generated by complementary mechanisms, resulting in significant weight loss, improved glycemic control, and lipid reduction, which contribute to disease management and its progression delay (Samms et al., 2020). In May 2022, the U.S. Food and Drug Administration (FDA) approved tirzepatide for type 2 diabetes (Mullard, 2022). Additionally, due to its weight-reducing effects, tirzepatide was approved in November 2023 for chronic weight management in obese or overweight adults with at least one weight-related condition, such as hypertension, type 2 diabetes, or hyperlipidemia. Owing to its metabolic effects, tirzepatide has a broad range of applications, including obstructive sleep apnea (Malhotra et al., 2024) and metabolic dysfunction-associated steatohepatitis with liver fibrosis (Loomba et al., 2024).

Given the remarkable efficacy and widespread use of tirzepatide in diabetes mellitus and weight control, understanding its potential for adverse drug events (ADEs) is crucial. In the SURPASS-1 to −5 trials (N = 6263), nausea (12%–24%), diarrhea (12%–22%), and vomiting (2%–13%) were the most common gastrointestinal ADEs associated with tirzepatide (Patel et al., 2024). The SURMOUNT-4 randomized clinical trial similarly identified mild to moderate gastrointestinal events as the most common adverse reactions (nausea [35.5%], diarrhea [21.1%], constipation [20.7%], and vomiting [16.3%]) (Aronne et al., 2024). Network meta-analyses underscore the need for safety concerns regarding drug use, particularly gastrointestinal events (Yao et al., 2024). A systematic review and meta-analysis of tirzepatide’s safety in patients with type 2 diabetes mellitus and obesity indicate that tirzepatide appears to be safe concerning pancreatitis risk (Zeng et al., 2023). However, the increased risk of gallbladder or biliary tract disease observed in clinical trials warrants further attention from physicians in clinical practice (Zeng et al., 2023). Information on tirzepatide’s efficacy and safety has been primarily documented through case reports, clinical trials, and meta-analyses. However, these studies often target specific populations or include relatively limited sample sizes and selection criteria, resulting in a lack of comprehensive safety data from large samples and real-world cohorts.

The FDA Adverse Event Reporting System (FAERS) database is an invaluable resource for post-marketing surveillance and early detection of drug safety issues. Regularly updated and publicly available for download on the FDA website, it contains authentic adverse event reports from various sources, including healthcare professionals, consumers, and manufacturers’ records. This study aims to assess tirzepatide’s safety through post-marketing surveillance and provide a comprehensive and valuable reference for its real-world safety.

## Methods

### Data sources and pre-processing

We conducted a retrospective pharmacovigilance analysis using the FAERS database, a publicly accessible repository of all self-reported adverse events documented by the FDA. The FAERS database currently houses over 10 million case reports. The FAERS database is composed of seven datasets: demographic and administrative details (DEMO), drug-related information (DRUG), adverse drug reaction details (REAC), patient outcomes (OUCT), sources of reports (RPSR), drug therapy start and end dates (THER), and indications for drug use (INDI) (Shu et al., 2022a). For this study, we searched the FAERS database for all tirzepatide cases since FDA approval to examine further. Key details such as patient demographic characteristics (age, sex, weight), reporting country, type of reporter, indication, suspected drug, adverse drug events, and patient prognosis were retrieved and analyzed.

To collect reports of ADEs related to tirzepatide, we extracted data from 1 April 2022 (Q2 2022) to 31 March 2024 (Q1 2024). Because FAERS does not use a uniform drug coding system, we used generic name (TIRZEPATIDE) and brand names (MOUNJARO, ZEPBOUND) to identify ADE reports associated with tirzepatide. Given that the database is updated quarterly, duplication of previously published reports is inevitable. To enhance the reliability of our findings, we adhered to FDA-recommended criteria: when CASEIDs were identical, the most recent FDA_DT was selected; when CASEIDs and FDA_DTs were identical, the higher PRIMARYID was chosen. ADEs were classified according to the top-level classification of the Medical Dictionary for Regulatory Activities (MedDRA, version 26.0) using the System Organ Class (SOC) terminology. SOC refers to the highest level of classification in medical terminology, used to organize and categorize medical information based on human systems or organs (Wu et al., 2019). To improve result accuracy and minimize the impact of confounding factors, we retained only the role codes for adverse events attributed to the primary suspect (PS) drug.

### Data analysis

We employed disproportionality methods, including the Reporting Odds Ratio (ROR), Proportional Reporting Ratio (PRR), Bayesian Confidence Propagation Neural Network (BCPNN), and Multi-Item Gamma Poisson Shrinker (MGPS) methods to detect signal strength (Zou et al., 2024). Frequentist methods, such as ROR and PRR, are highly sensitive, easy to compute, and effective for identifying common signals. However, they are prone to generating false-positive signals when the number of reports is low (Jiang et al., 2024). In contrast, Bayesian methods, including BCPNN and MGPS, reduce false positives by incorporating statistical corrections and probability distributions, making them more suitable for detecting rare events. Nevertheless, Bayesian approaches tend to have delayed signal detection and are computationally more complex (Nomura et al., 2015). By simultaneously utilizing multiple algorithms, we leveraged the strengths of different approaches and extended the detection range. This comprehensive approach allowed us to thoroughly review results from various perspectives to identify robust and reliable safety signals. All algorithms are based on a 2 × 2 contingency table (as shown in Table 1). Preferred terms (PTs) serve as the core terminology in MedDRA, providing standardized and precise descriptions of medical events and concepts (Brown, 2024). To ensure result reliability, only PTs that satisfied all four algorithms simultaneously were considered significant signals. ADEs related to drug indications were excluded, and unexpected signals not included in the drug labeling were also deemed important signals. To reduce the likelihood of all false-positive results occurring (reducing the probability of Type I errors), we used the Bonferroni method to correct for multiple testing of *P*-values (Curtin and Schulz, 1998). The formula for Bonferroni: Bonferroni = *P*/n, where *P* is the original threshold and n is the total number of tests.

**TABLE 1:** Methods, formulas, and thresholds for Reporting Odds Ratio (ROR), Proportional Reporting Ratio (PRR), Bayesian Confidence Propagation Neural Network (BCPNN), and Empirical Bayesian Geometric Mean (EBGM). (a) Count of reports featuring both the specified drug and target adverse events; (b) number of reports involving other adverse drug events alongside the specified drug; (c) reports of target adverse drug events involving other drugs; (d) reports encompassing other drugs and non-targeted adverse drug events. 95% CI: 95% confidence interval; N: number of reports; χ2: chi-squared; IC: information component; IC025: lower limit of 95% CI of the IC; E (IC): IC expectations; V(IC): variance of IC; EBGM05: lower limit of 95% CI of EBGM.

Algorithms	Equation	Criteria
ROR	ROR=ad/bc 95%CI=e⁡lnROR±1.96(1/a+1/b+1/c+1/d)^ 0.5	lower limit of 95% CI > 1,
PRR	$\text{PRR}=a\left(c+d\right)/c\left(a+b\right)$ χ2=ad−bc)^ 2a+b+c+d/a+bc+da+cb+dIC=log⁡2aa+b+c+d/(a+ca+b	N ≥ 3 PRR ≥ 2, χ2 ≥ 4, N ≥ 3
BCPNN	95%CI=EIC ± 2VIC^ 0.5 $\text{EBGM}=a\left(a+b+c+d\right)/\left(a+c\right)/\left(a+b\right)$	IC025 > 0
MGPS	95%CI=e⁡lnEBGM±1.96(1/a+1/b+1/c+1/d)^ 0.5	EBGM05 > 2

### Time to onset (TTO) analysis

We defined the time to onset (TTO) of ADEs associated with tirzepatide as the period between the ADE occurrence date in the DEMO file (EVENT_DT) and the dosing initiation date in the THER file (START_DT). Cases with inaccurate or missing data and ADE start dates preceding the medication start date were excluded from the analysis. The Weibull distribution test determines the proportional change in the incidence of adverse events, indicating an increasing or decreasing risk over time (Shu et al., 2022b). We performed a comprehensive TTO assessment based on median, quartiles, extremes, and the Weibull distribution test.

### Statistical analysis

All data processing and statistical analyses were conducted using Microsoft Excel 2019 and R software (version 4.2.1). The “ggplot2” package in R software was employed for data visualization.

## Results

### Descriptive characteristics

In this study, 3,445,594 reported cases were collected from the FAERS database during the study period (Q2 2022-Q1 2024), resulting in 37,827 tirzepatide-associated ADEs and 78,709 tirzepatide-associated PTs after the removal of duplicates. The demographic characteristics are shown in Table 2. Notably, there were more female patients reported (68.8%) compared to male patients (19%). Among the known age information reported, patients aged 18–65 years had the highest number of reports at 16,842. Unfortunately, the vast majority of reported cases lacked weight information. The United States had the most recorded information (98.9%). The majority of reports were submitted by consumers (93.9%). Of the reported outcomes, other serious outcomes (n = 1283) were the most frequently recorded, followed by hospitalization-initial or prolonged (n = 987), death (n = 94), and life-threatening (n = 62). Unspecified indications for the use of tirzepatide were recorded in 57.3% of cases, with type 2 diabetes mellitus (20.8%) being the most commonly reported indication. Figure 2A shows the annual distribution of tirzepatide-related ADE reports.

**TABLE 2:** Demographic characteristics of ADEs reported in the FAERS database (April 2022-March 2024) with tirzepatide as the primary suspect drug. ADEs: adverse drug events.

Characteristics	Case number, n	Case proportion, %
Gender		
Female	26,042	68.8%
Male	7176	19.0%
Unknown	4609	12.2%
Age		
<18	9	0.0%
18-65	16,842	44.5%
>65	3065	8.2%
Unknown	17,911	47.3%
Weight		
<50	17	0.0%
50-100	419	1.1%
>100	281	0.8%
Unknown	37,110	98.1%
Reported Countries (top three)		
United States	37,401	98.9%
Japan	243	0.6%
United Arab Emirates	103	0.3%
Reported person		
Health professionals	2281	6.0%
Consumer	35,517	93.9%
Unknown	29	0.1%
Outcome		
Hospitalization-initial or prolonged	987	2.6%
Life-threatening	62	0.2%
Disability	56	0.1%
Congenital anomaly	4	0.0%
Death	94	0.2%
Other serious outcome	1283	3.4%
Required intervention	51	0.1%
Unknown	35,505	93.3%
Indication (top five)		
Product used for unknown indication	21,693	57.3%
Type 2 diabetes mellitus	7891	20.8%
Weight decreased	1967	5.1%
Diabetes mellitus	1934	5.1%
Glucose tolerance impaired	1339	3.5%

**FIGURE 1:**
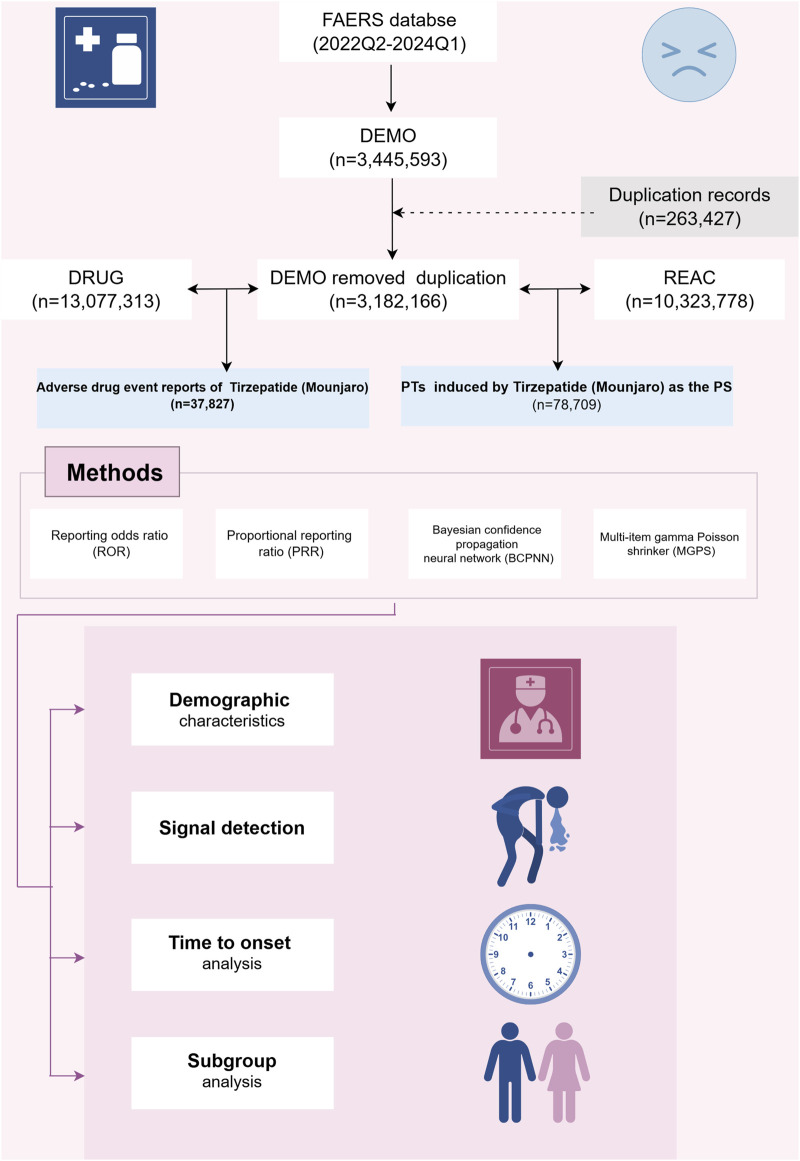
Flowchart of the entire study and presentation of the main findings.

**FIGURE 2:**
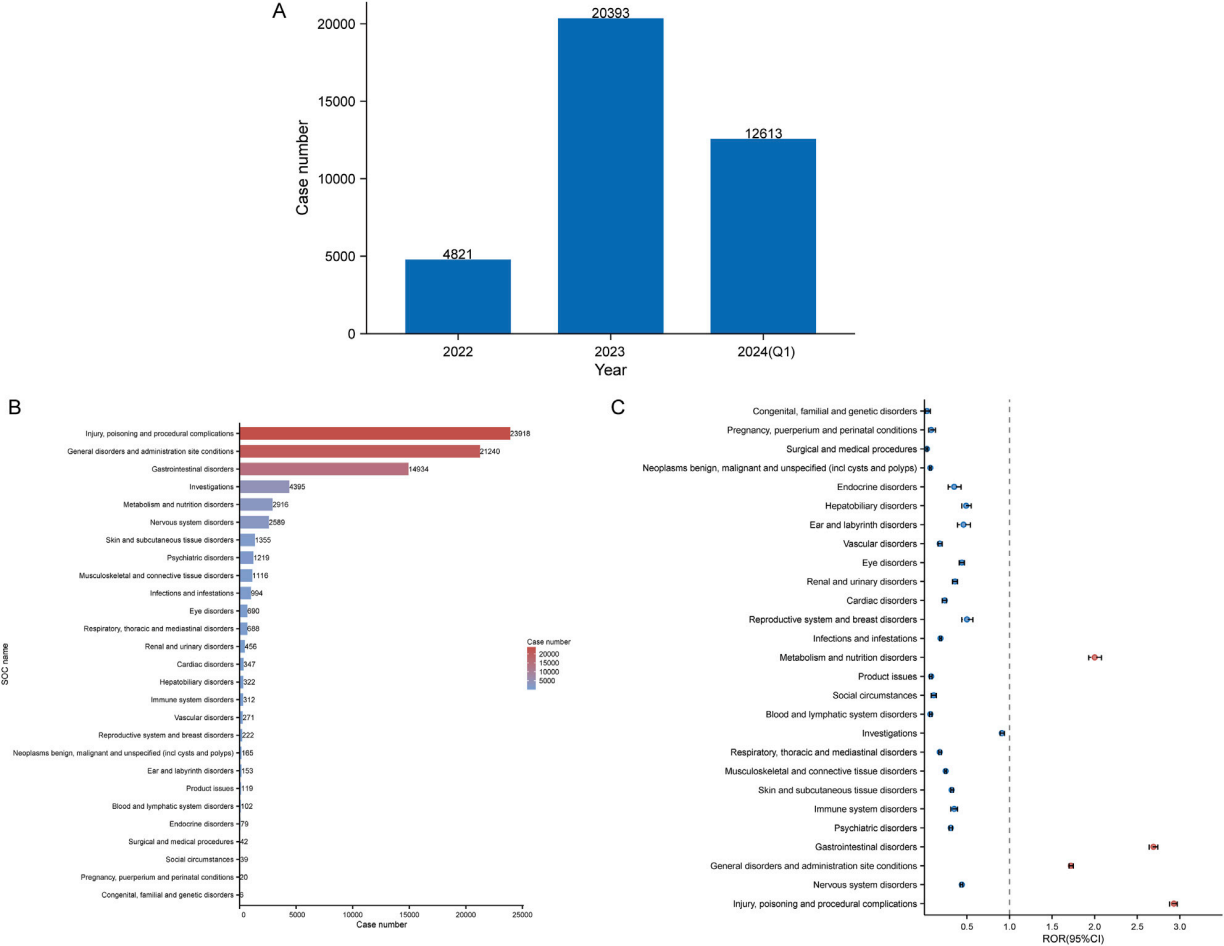
Signals detection at the SOC level. **(A)** Distribution of ADEs of tirzepatide from 2022 to the first quarter of 2024 (2024 Q1). **(B)** Bar chart displaying the reported cases of ADEs at each SOC level. **(C)** Signals detection at the SOC level, with ROR values and their 95% confidence intervals (95% CI) visualized. SOCs with positive signal values are labeled for distinction. FAERS: Food and Drug Administration (FDA) Adverse Event Reporting System; ADEs: adverse drug events; SOC: System Organ Class; ROR: reporting odds ratio.

### Signal detection of system organ class

Table 3 shows the signal intensity and number of case reports associated with tirzepatide for each SOC. According to our statistical analysis, a total of 27 organ systems were affected by tirzepatide-related ADEs. The ranking of the number of ADEs for tirzepatide-related SOCs is presented in Figure 2B. The top three SOCs were injury, poisoning and procedural complications (n = 23,918), general disorders and administration site conditions (n = 21,240), and gastrointestinal disorders (n = 14,934). Significant SOCs for which at least one of the four methods of disproportionality analysis met the criteria were injury, poisoning and procedural complications (SOC code: 10000044), general disorders and administration site conditions (SOC code: 10017581), gastrointestinal disorders (SOC code: 10000050), and metabolism and nutrition disorders (SOC code: 10065941). Figure 2C shows the ROR of tirzepatide-related SOC signal intensity and its 95% confidence interval.

**TABLE 3:** Signal intensity of ADE reports concerning tirzepatide at the system organ class (SOC) level within the FAERS database.

System Organ Class	SOC code	Case reports	ROR(95%CI)	PRR(χ2)	EBGM(EBGM05)	IC(IC025)
Injury, poisoning and procedural complications	10000044	23918	2.93(2.88-2.97)	2.34(20793.40)	2.32(2.28)	1.21(-0.45)
General disorders and administration site conditions	10017581	21240	1.72(1.70-1.75)	1.53(4651.50)	1.52(1.50)	0.61(-1.06)
Gastrointestinal disorders	10000050	14934	2.69(2.64-2.74)	2.37(12592.71)	2.34(2.30)	1.23(-0.44)
Investigations	10063264	4395	0.91(0.89-0.94)	0.92(34.19)	0.92(0.89)	-0.12(-1.79)
Metabolism and nutrition disorders	10065941	2916	2.00(1.93-2.08)	1.96(1384.24)	1.95(1.88)	0.96(-0.70)
Nervous system disorders	10000346	2589	0.44(0.42-0.45)	0.45(1816.94)	0.46(0.44)	-1.13(-2.80)
Skin and subcutaneous tissue disorders	10058820	1355	0.32(0.31-0.34)	0.33(1890.14)	0.34(0.32)	-1.57(-3.24)
Psychiatric disorders	10082331	1219	0.31(0.29-0.33)	0.32(1818.62)	0.32(0.31)	-1.63(-3.29)
Musculoskeletal and connective tissue disorders	10074599	1116	0.25(0.24-0.26)	0.26(2479.63)	0.26(0.25)	-1.94(-3.60)
Infections and infestations	10060921	994	0.19(0.18-0.20)	0.20(3433.81)	0.20(0.19)	-2.33(-3.99)
Eye disorders	10000173	690	0.44(0.41-0.47)	0.44(485.87)	0.45(0.41)	-1.16(-2.83)
Respiratory, thoracic and mediastinal disorders	10051545	688	0.18(0.17-0.20)	0.19(2504.30)	0.19(0.18)	-2.39(-4.06)
Renal and urinary disorders	10073515	456	0.36(0.33-0.39)	0.36(525.34)	0.36(0.33)	-1.47(-3.13)
Cardiac disorders	10077162	347	0.24(0.21-0.26)	0.24(848.59)	0.24(0.22)	-2.05(-3.72)
Hepatobiliary disorders	10071634	322	0.49(0.44-0.55)	0.50(165.17)	0.50(0.45)	-1.00(-2.67)
Immune system disorders	10000206	312	0.35(0.31-0.39)	0.35(381.51)	0.35(0.31)	-1.51(-3.18)
Vascular disorders	10000358	271	0.18(0.16-0.21)	0.19(987.57)	0.19(0.17)	-2.42(-4.09)
Reproductive system and breast disorders	10087591	222	0.50(0.44-0.57)	0.50(112.62)	0.50(0.44)	-1.00(-2.67)
Neoplasms benign, malignant and unspecified (incl cysts and polyps)	10068532	165	0.07(0.06-0.08)	0.07(2038.98)	0.07(0.06)	-3.79(-5.46)
Ear and labyrinth disorders	10063559	153	0.46(0.39-0.54)	0.46(96.77)	0.46(0.39)	-1.11(-2.78)
Product issues	10078577	119	0.08(0.06-0.09)	0.08(1337.37)	0.08(0.06)	-3.68(-5.35)
Blood and lymphatic system disorders	10073485	102	0.07(0.06-0.09)	0.08(1179.64)	0.08(0.06)	-3.72(-5.39)
Endocrine disorders	10080230	79	0.35(0.28-0.43)	0.35(96.50)	0.35(0.28)	-1.52(-3.18)
Surgical and medical procedures	10059486	42	0.03(0.02-0.04)	0.03(1198.63)	0.03(0.02)	-4.90(-6.56)
Social circumstances	10000209	39	0.11(0.08-0.14)	0.11(296.05)	0.11(0.08)	-3.23(-4.90)
Pregnancy, puerperium and perinatal conditions	10084854	20	0.08(0.05-0.13)	0.08(198.90)	0.09(0.05)	-3.56(-5.22)
Congenital, familial and genetic disorders	10000002	6	0.03(0.01-0.07)	0.03(189.51)	0.03(0.01)	-5.05(-6.72)

### Signal detection of preferred terms

**FIGURE 3:**
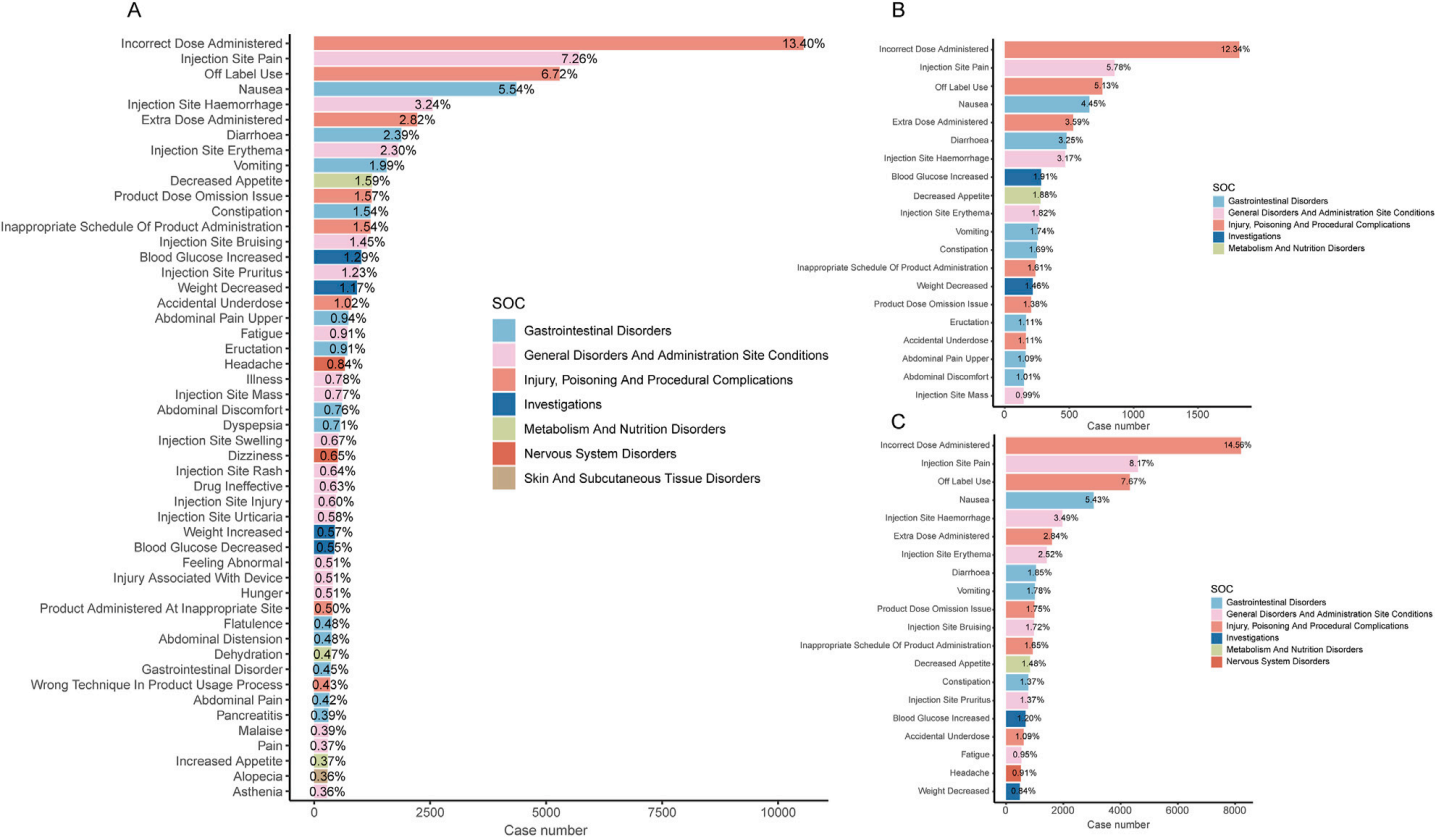
**(A)** Bar chart displaying the case number and frequency of the top 50 preferred terms (PTs) for tirzepatide. **(B)** Bar chart displaying the case number and frequency of the top 20 preferred terms (PTs) for tirzepatide in males. **(C)** Bar chart displaying the case number and frequency of the top 20 preferred terms (PTs) for tirzepatide in females.

**FIGURE 4:**
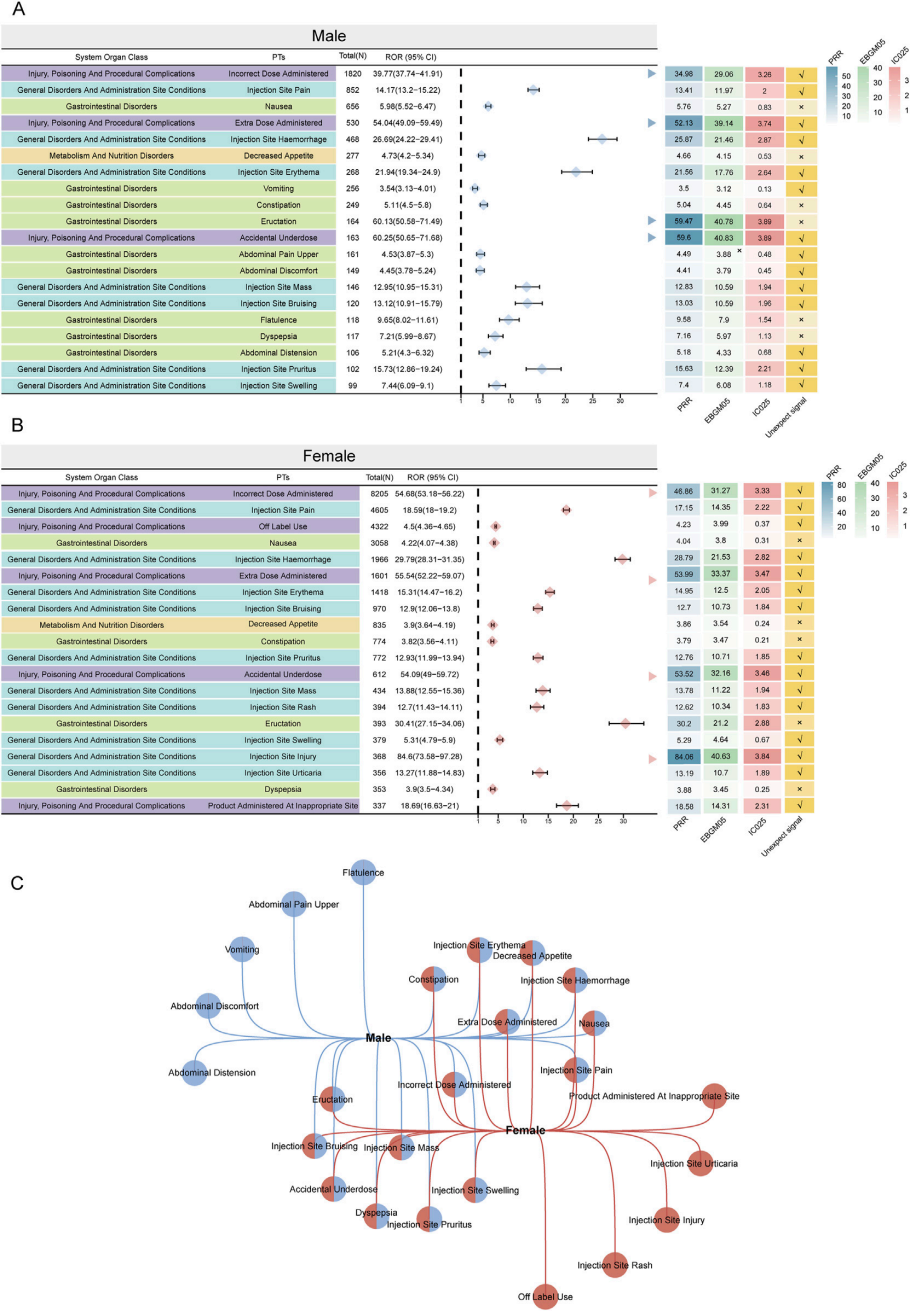
The twenty most prominent ADE signals at the PT level, categorized by sexsubgroups, are indicated. Arrows in **(A, B)** denote instances where the lower boundary of the 95% CI of the ROR surpasses 25. **(C)** Overlap of the top twenty signals in both subgroups. PRR: proportional reporting ratio; EBGM05: lower limit of 95% CI of EBGM; IC025: lower limit of 95% CI of the IC; ADE: adverse drug event; PT: preferred term; ROR: reporting odds ratio; CI: confidence interval.

Figure 3A shows the top 50 PTs with the highest percentage of tirzepatide, sorted by the number of case reports, involving 7 SOCs. The top five PTs were incorrect dose administered (13.4%), injection site pain (7.26%), off-label use (6.72%), nausea (5.54%), and injection site hemorrhage (3.24%). Figures 3B, C show the top 20 PTs with the highest percentage of tirzepatide use in males and females, respectively. Among these, nervous system disorders were uniquely significant in females. After excluding PTs as possible indications for tirzepatide, we identified 100 significantly disproportionate PTs that met the criteria of all four disproportionality analysis methods and the Bonferroni test for correction (Supplementary Table S1). And, considering the results of the most stringent EBGM algorithm, we ranked these PTs and listed the top 50 PTs in Table 4. Concerningly, unexpected significant ADEs were revealed, with the top six strongest signal ADEs ranked by the EBGM being injection site coldness (n = 86, ROR 155.64, PRR 155.47, IC 71.39, EBGM 6.16), injection site injury (n = 86, ROR 155.64, PRR 155.47, IC 71.39, EBGM 6.16), accidental underdose (n = 470, ROR 58.11, PRR 57.52, IC 40.2, EBGM 5.33), extra dose administered (n = 2222, ROR 49.7, PRR 48.32, IC 35.51, EBGM 5.15), injection site laceration (n = 19, ROR 46.67, PRR 46.66, IC 34.61, EBGM 5.11), and starvation ketoacidosis (n = 7, ROR 45.56, PRR 45.56, IC 34.01, EBGM 5.09). Sensitivity analyses incorporating sex were conducted to improve the reliability of the results, considering the potentially confounding effect of baseline information on the disproportionality analysis results. Figures 4A, B depict the top 20 PTs ranked by the number of reports for males and females, respectively. As Figure 4C shows, for further comparison, we took the intersection between them. Abdominal distension, abdominal discomfort, vomiting, abdominal pain upper, and flatulence have higher reporting ranks in males, while off-label use, injection site rash, injection site injury, injection site urticaria, and product administered at an inappropriate site have higher reporting ranks in females.

**TABLE 4:** Top 50 signal intensity of ADEs of tirzepatide ranked by EBGM at the PT level. ADEs: adverse drug events; EBGM: Empirical Bayesian Geometric Mean; PTs: preferred terms.

System Organ Class	PTs	Case reports	ROR(95% CI)	PRR(χ2 )	IC(IC025)	EBGM(EBGM05)
General Disorders And Administration Site Conditions	Injection Site Coldness^*^	86	55.64(113.79-212.8	155.47(6014.87)	71.39(54.93)	6.16(4.48)
General Disorders And Administration Site Conditions	Injection Site Injury^*^	470	85.59(76.18-96.16)	85.09(23620)	51.85(47.03)	5.7(4.03)
Injury, Poisoning And Procedural Complications	Accidental Underdose^*^	803	58.11(53.46-63.16)	57.52(30938.02)	40.2(37.49)	5.33(3.66)
Injury, Poisoning And Procedural Complications	Extra Dose Administered^*^	2222	49.7(47.31-52.21)	48.32(75157.31)	35.51(34.08)	5.15(3.48)
General Disorders And Administration Site Conditions	Injection Site Laceration^*^	19	46.67(27.63-78.83)	46.66(624.95)	34.61(22.32)	5.11(3.41)
Metabolism And Nutrition Disorders	Starvation Ketoacidosis^*^	7	45.56(19.26-107.75	45.56(225.96)	34.01(16.55)	5.09(3.33)
Gastrointestinal Disorders	Eructation	716	43.63(40.09-47.5)	43.25(22185.95)	32.71(30.47)	5.03(3.36)
Injury, Poisoning And Procedural Complications	Incorrect Dose Administered^*^	10548	47.12(46.04-48.22)	40.94(314002.68)	31.38(30.78)	4.97(3.31)
General Disorders And Administration Site Conditions	Injection Site Paraesthesia^*^	70	37.84(29-49.38)	37.81(1943.79)	29.52(23.63)	4.88(3.21)
Investigations	Blood Calcitonin Increased^*^	4	37.19(12.24-112.99	37.19(109.56)	29.15(11.5)	4.87(3.07)
Product Issues	Product Tampering^*^	56	35.58(26.47-47.82)	35.56(1477.25)	28.14(21.98)	4.81(3.14)
General Disorders And Administration Site Conditions	Hunger^*^	402	31.45(28.2-35.08)	31.29(9506.21)	25.42(23.2)	4.67(3)
General Disorders And Administration Site Conditions	Injection Site Haemorrhage^*^	2554	30.49(29.19-31.85)	29.53(57459.39)	24.26(23.39)	4.6(2.93)
Metabolism And Nutrition Disorders	Starvation^*^	26	25.84(16.96-39.36)	25.83(517.9)	21.72(15.27)	4.44(2.76)
General Disorders And Administration Site Conditions	Injection Site Dermatitis^*^	4	21.69(7.53-62.53)	21.69(67.68)	18.74(7.73)	4.23(2.48)
Metabolism And Nutrition Disorders	Food Craving^*^	106	20.5(16.7-25.16)	20.47(1696.42)	17.82(15.02)	4.16(2.49)
General Disorders And Administration Site Conditions	Thirst Decreased^*^	13	18.8(10.51-33.64)	18.8(191.45)	16.55(10.18)	4.05(2.36)
General Disorders And Administration Site Conditions	Injection Site Erythema^*^	1813	18.65(17.75-19.6)	18.25(25957.95)	16.13(15.47)	4.01(2.35)
Neoplasms Benign, Malignant And Unspecified (Incl Cysts And Polyps)	Medullary Thyroid Cancer	7	17.19(7.82-37.82)	17.19(94.29)	15.3(7.91)	3.94(2.23)
General Disorders And Administration Site Conditions	Injection Site Pain^*^	5717	18.46(17.94-18.99)	17.19(77376.77)	15.3(14.94)	3.94(2.27)
Gastrointestinal Disorders	Impaired Gastric Emptying^*^	181	16.6(14.22-19.38)	16.57(2349.1)	14.81(13.01)	3.89(2.22)
General Disorders And Administration Site Conditions	Injection Site Urticaria^*^	455	16.52(14.98-18.21)	16.43(5856.03)	14.7(13.54)	3.88(2.21)
Neoplasms Benign, Malignant And Unspecified (Incl Cysts And Polyps)	Follicular Thyroid Cancer	3	16.27(4.9-54.04)	16.27(38.22)	14.57(5.34)	3.87(2.11)
Investigations	Glycosylated Haemoglobin Decreased^*^	38	16.01(11.43-22.43)	16.01(476.11)	14.36(10.83)	3.84(2.17)
General Disorders And Administration Site Conditions	Injection Site Pruritus^*^	969	16.13(15.09-17.25)	15.95(12104.37)	14.32(13.53)	3.84(2.17)
General Disorders And Administration Site Conditions	Injection Site Irritation^*^	143	15.37(12.92-18.28)	15.34(1715.62)	13.83(11.96)	3.79(2.12)
General Disorders And Administration Site Conditions	Injection Site Rash^*^	503	15.43(14.06-16.93)	15.34(6033.3)	13.83(12.79)	3.79(2.12)
Metabolism And Nutrition Disorders	Lack Of Satiety^*^	4	15.31(5.43-43.16)	15.31(47.88)	13.81(5.8)	3.79(2.06)
General Disorders And Administration Site Conditions	Injection Site Macule^*^	3	15.02(4.55-49.62)	15.02(35.2)	13.57(4.99)	3.76(2.01)
Metabolism And Nutrition Disorders	Increased Appetite	292	15(13.28-16.93)	14.95(3409.3)	13.51(12.21)	3.76(2.09)
General Disorders And Administration Site Conditions	Injection Site Thrombosis^*^	5	14.79(5.87-37.3)	14.79(57.73)	13.38(6.17)	3.74(2.03)
General Disorders And Administration Site Conditions	Injection Site Bruising^*^	1141	14.98(14.08-15.93)	14.77(13173.7)	13.37(12.7)	3.74(2.07)
General Disorders And Administration Site Conditions	Injection Site Hypersensitivity^*^	51	13.75(10.3-18.35)	13.74(545.12)	12.53(9.84)	3.65(1.98)
Injury, Poisoning And Procedural Complications	Intercepted Product Selection Error^*^	9	13.47(6.78-26.75)	13.47(94.12)	12.3(6.92)	3.62(1.93)
Injury, Poisoning And Procedural Complications	Hypobarism^*^	3	13.47(4.1-44.21)	13.47(31.37)	12.3(4.55)	3.62(1.88)
General Disorders And Administration Site Conditions	Injection Site Mass^*^	608	13.31(12.24-14.47)	13.21(6235.49)	12.09(11.27)	3.6(1.93)
Injury, Poisoning And Procedural Complications	Product Administered At Inappropriate Site	393	12.37(11.15-13.72)	12.31(3732.03)	11.33(10.39)	3.5(1.84)
Investigations	Blood Glucose Decreased	433	12.19(11.04-13.45)	12.13(4046.4)	11.18(10.29)	3.48(1.82)
General Disorders And Administration Site Conditions	Injury Associated With Device^*^	402	12.18(10.99-13.49)	12.12(3754.96)	11.18(10.26)	3.48(1.82)
Product Issues	Suspected Product Tampering^*^	11	11.74(6.33-21.76)	11.74(99.1)	10.85(6.47)	3.44(1.75)
Investigations	Gastric Ph Decreased^*^	13	11.51(6.53-20.3)	11.51(114.64)	10.66(6.63)	3.41(1.73)
Investigations	Blood Insulin Increased^*^	8	11.2(5.44-23.06)	11.2(68.4)	10.39(5.68)	3.38(1.69)
General Disorders And Administration Site Conditions	Injection Site Discomfort^*^	135	10.14(8.51-12.08)	10.12(1029.92)	9.46(8.17)	3.24(1.58)
Nervous System Disorders	Allodynia^*^	17	9.88(6.03-16.18)	9.88(126.09)	9.25(6.12)	3.21(1.53)
General Disorders And Administration Site Conditions	Injection Site Scar^*^	33	9.61(6.75-13.69)	9.61(237.05)	9.02(6.71)	3.17(1.5)
Injury, Poisoning And Procedural Complications	Foreign Body^*^	5	9.57(3.86-23.74)	9.57(35.75)	8.98(4.2)	3.17(1.47)
Investigations	Pancreatic Enzymes Increased^*^	14	9.54(5.55-16.42)	9.54(99.73)	8.96(5.69)	3.16(1.49)
General Disorders And Administration Site Conditions	Injection Site Scab^*^	8	9.38(4.58-19.23)	9.38(55.87)	8.82(4.84)	3.14(1.45)
General Disorders And Administration Site Conditions	Injection Site Inflammation^*^	52	9.07(6.84-12.01)	9.06(348.66)	8.54(6.75)	3.09(1.42)
General Disorders And Administration Site Conditions	Injection Site Hypoaesthesia^*^	18	9.05(5.61-14.59)	9.05(120.45)	8.52(5.71)	3.09(1.42)

^*^ Signals not listed on the drug label are marked with asterisks (*).

### TTO analysis of tirzepatide-related ADEs from the overall and SOC levels

**FIGURE 5:**
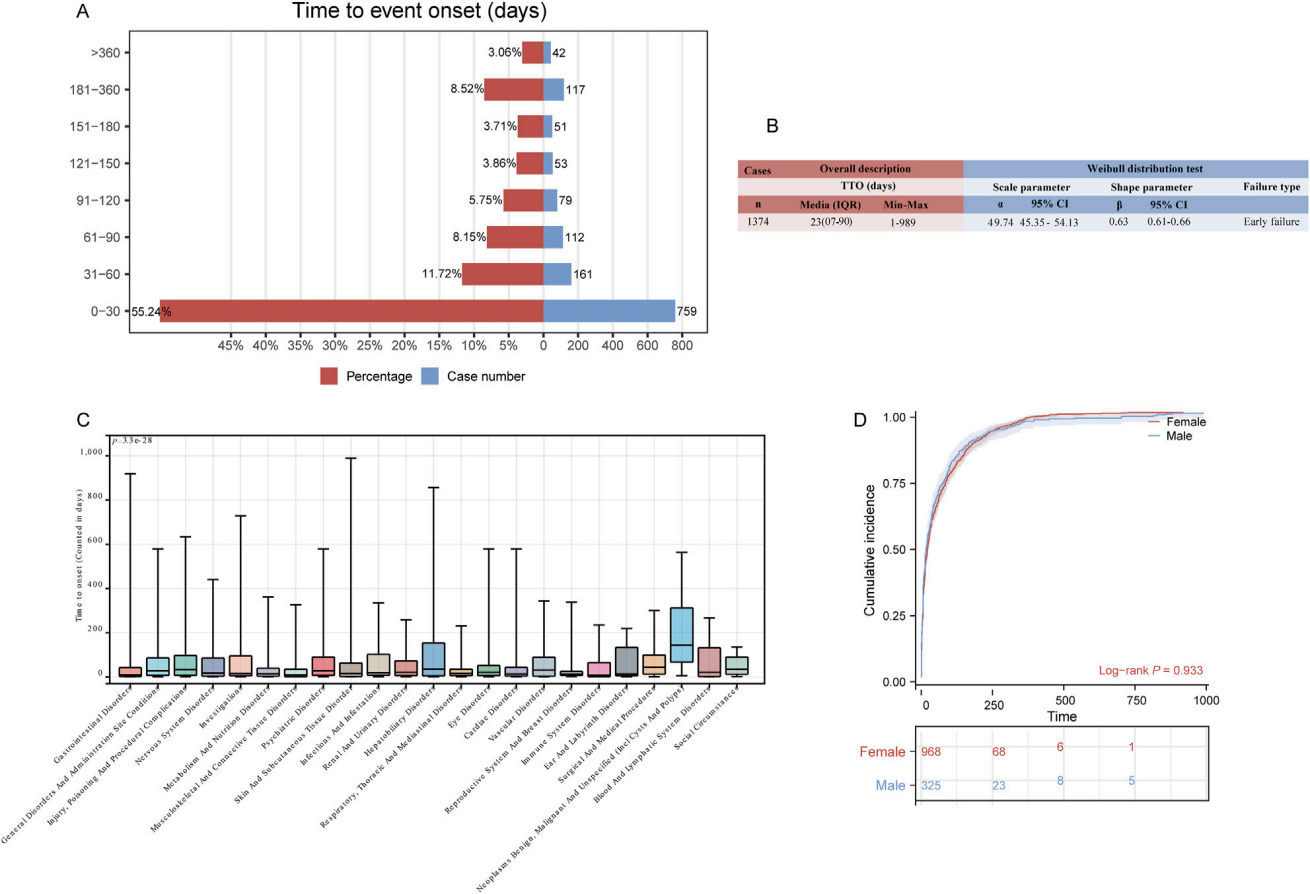
Time to onset (TTO) analysis (counted in days) of tirzepatide-related ADEs. **(A)** Bar charts illustrating the quantity and proportion of TTO reports within varying time intervals. **(B)** Weibull distribution test for TTO analysis. **(C)** Box plot displaying the TTO of ADEs at the SOC level. Bold white bar within the stick: median TTO; lower end of the stick: 1/4 quantile of the TTO; upper end of the stick: 3/4 quantile of the TTO. **(D)** Comparison of cumulative incidences of ADEs between males and females. IQR: interquartile range; Min: minimum; Max: maximum.

A total of 1,374 (3.6%) reported adverse events included comprehensive and accurate details about the time of occurrence. As shown in Figure 5A, more than half of the cases occurred within the first month (n = 759, 55.24%). Of note, our data showed that 3.06% of AEs occurred after 1 year of tirzepatide treatment. The median time to onset of ADEs is 23 days, with an interquartile range [IQR] of 7–90 days. The shape parameter (β) was 0.63, and the upper limit of its 95% confidence interval (CI) was 0.66, indicating a decrease in the incidence of ADEs over time (Figure 5B). To assess the time to onset of ADEs in further detail, we analyzed TTO at the SOC level (Figure 5C; Supplementary Table S2). The longest-onset SOC, neoplasms benign, malignant, and unspecified (incl cysts and polyps), had a median onset of 144 days (IQR 67–312 days). In contrast, immune system disorders had the shortest median onset times at 8 days (IQR 1–65 days). The Kaplan-Meier curves in Figure 5D show the cumulative incidence of tirzepatide ADEs in the sex subgroups, with no significant differences observed.

## Discussion

In this study, we conducted a quantitative assessment of tirzepatide’s safety through a comprehensive analysis of real-world data from the FAERS database. This evaluation was performed from both an overall population perspective and a sex-specific perspective, aiming to balance the drug’s benefits and risks and provide informed guidance for clinical decision-making.

Tirzepatide-related ADEs were more frequently observed in females (68.8%) compared to males (19%). This discrepancy may be attributed to the drug’s relatively short market presence and the limited availability of data that require further observation over time. Additionally, the primary reporters of these ADEs were consumers, and social factors may contribute to females being more proactive in reporting adverse reactions (Lee et al., 2023). The epidemiologic characteristics of the obese population also support these findings (Jaacks et al., 2019; NCD Risk Factor, 2024). ADEs were more prevalent in individuals aged 18-65 who received tirzepatide. Unfortunately, over 90% of the reports lacked detailed information on patients’ weight and outcomes. The U.S was the predominant reporting country (98.9%), likely because it was the first to authorize the drug for marketing. The annual analysis report shows more than half of the ADEs were reported in the first quarter of 2024 than in the entire year of 2023. These results underscore the widespread clinical use and efficacy of tirzepatide and highlight the importance of enhancing the detection of tirzepatide-related ADEs.

The three most common SOCs identified in our study with tirzepatide were injury, poisoning and procedural complications, general disorders and administration site conditions, and gastrointestinal disorders. Injury, poisoning, and procedural complications were not mentioned in the drug labeling, but the other two were consistent with the documented safety information. The results of the disproportionality analysis showed four significant signals at the SOC level, some of which were explicitly mentioned in the drug labeling, validating the reliability of our findings. The most common ADEs reflected reporter concerns about drug use. Incorrect dose administered, injection site pain, off-label use, and nausea were the top four PTs with the highest percentage of tirzepatide for both males and females. Notably, incorrect dose administered and off-label use were unexpected PTs, emphasizing the importance of standardizing medication use and increasing restrictions on prescriptions by healthcare professionals. Headache (n = 511, 0.91%) is a common nervous system disorder among females. Many clinical studies have reported adverse effects of headaches (Frias et al., 2018; Rosenstock et al., 2021; Garvey et al., 2023), but the relationship with sex is unknown. Further studies on the relationship between tirzepatide and headaches may be needed.

The signals with strongest signal values included injection site coldness, injection site injury, accidental underdose, extra dose administered, injection site laceration, and starvation ketoacidosis. The SURPASS trial indicated a higher incidence of injection site responses than placebo, which were mild to moderate (Rosenstock et al., 2021; Dahl et al., 2022). Injection reactions to tirzepatide are also mentioned in the drug label but not in detail. Our data provides a detailed list of the number of cases and signal values of injection ADEs associated with tirzepatide, aiding doctors in detecting these ADEs. Notably, starvation ketoacidosis exhibited a strong correlation, with a significant signal strength of ROR 45.56 (19.26-107.75), PRR 45.56, IC 34.01, EBGM 5.09. There are two known cases of tirzepatide-induced ketoacidosis (Mercer et al., 2023; Iqbal et al., 2024). Both cases were female non-diabetic obese patients, with starvation ketoacidosis being the likely mechanism. Ketoacidosis is serious, and physicians should be aware of this complication and alert for early symptoms.

Gastrointestinal events were the most common treatment-emergent ADEs in several clinical trials, and most were mild to moderate in intensity (Frias et al., 2018; Ludvik et al., 2021; Rosenstock et al., 2021). Through a real-world analysis of tirzepatide, we identified several significant gastrointestinal ADEs, including nausea (n = 4358, ROR 5.20, PRR 4.97, IC 2.27, EBGM 4.82), constipation (n = 1215, ROR 4.48, PRR 4.43, IC 2.11, EBGM 4.32), eructation (n = 716, ROR 43.63, PRR 43.25, IC 32.71, EBGM 5.03), dyspepsia (n = 559, ROR 5.12, PRR 5.09, IC 2.30, EBGM 4.93), flatulence (n = 380, ROR 6.00, PRR 5.98, IC 2.53, EBGM 5.76), and pancreatitis (n = 307, ROR 7.18, PRR 7.16, IC 2.77, EBGM 6.84), consistent with previous clinical studies and drug labeling. A randomized, parallel-group, multicenter, phase 3 trial found that tirzepatide caused pancreatitis (5 mg n = 3, 10 mg n = 2, 15 mg n = 1) in patients with type 2 diabetes mellitus (Del Prato et al., 2021), and another double-blind, randomized, multicenter, phase 3 trial found that tirzepatide also caused pancreatitis (15 mg n = 2) in obese patients with type 2 diabetes mellitus (Garvey et al., 2023). It is important to note that clinical trials of overweight or obesity and obstructive sleep apnea and obesity also found adverse event reports of pancreatitis (Kadowaki et al., 2022; Wadden et al., 2023; Malhotra et al., 2024). These studies were constrained by relatively limited sample sizes and specific selection criteria. Based on our findings, vigilance regarding the association between tirzepatide and pancreatitis is needed, which may require further experimental verification. We recommend observing patients for nausea, vomiting, and abdominal pain during dosing, monitoring amylase and lipase levels in the patient’s blood and urine for early recognition, and conducting abdominal computed tomography scans if necessary.

We also identified unexpected signals, such as impaired gastric emptying (n = 181, ROR 16.60, PRR 16.57, IC 3.89, EBGM 14.81), abnormal gastrointestinal sounds (n = 37, ROR 5.28, PRR 5.27, IC 2.35, EBGM 5.11), and projectile vomiting (n = 22, ROR 8.09, PRR 8.09, IC 2.94, EBGM 7.67). Hypoglycemia is a low blood sugar condition that occurs during medication and can lead to discomfort and even life-threatening situations. Fortunately, fewer severe hypoglycemic events have been reported in prior clinical studies, but a higher incidence was observed in patients receiving background sulfonylurea and glinides. Therefore, regular monitoring of blood glucose during the use of hypoglycemic drugs is recommended to help prevent hypoglycemia.

For ADEs of special interest, both medullary thyroid cancer (n = 7, ROR 17.19, PRR 17.19, IC 15.3, EBGM 3.94) and follicular thyroid cancer (n = 3, ROR 16.27, PRR 16.27, IC 14.57, EBGM 3.87) had higher EBGM values. Based on the results of a rat study, a black box warning was issued in the United States in the prescribing information for tirzepatide, alerting patients to the risk of thyroid C-cell tumors and recommending further evaluation in patients with significantly elevated calcitonin levels or those found to have thyroid nodules. Our study identified diabetic retinopathy (n = 15, ROR 5.45, PRR 5.45, IC 2.40, EBGM 5.27) and retinopathy (n = 12, ROR 3.63, PRR 3.63, IC 1.83, EBGM 3.56). Although one study showed that tirzepatide did not affect the risk of diabetic retinopathy in patients with type 2 diabetes mellitus, however, there is still a need for large-scale randomized controlled trials are needed to assess the long-term safety of tirzepatide with respect to the risk of diabetic retinopathy (Popovic et al., 2024).

A Korean study on antidiabetic drugs revealed significant sex differences, with females reporting more ADEs than males (Joung et al., 2020). Our study similarly found significant sex differences in the reported baseline profiles, prompting a further comparison of ADEs between sexes. For males, the top three case-number significant PTs were incorrect dose administered, injection site pain, and nausea, whereas for females, they were incorrect dose administered, injection site pain, and off-label use. Interestingly, unique ADEs in males were more frequently related to gastrointestinal disorders, while in females, they were associated with general disorders and administration site conditions. Males are often linked with unhealthy lifestyles, such as poor diet, stress, physical inactivity, smoking, and excessive alcohol consumption, leading to a higher prevalence of non-malignant upper gastrointestinal diseases (Bai et al., 2024). In contrast, females may experience more distress from externally visible drug ADEs due to higher societal expectations and internalized pressures regarding appearance, making them more likely to report such ADEs (Lee et al., 2023). Skin hypersensitivity, an allergic manifestation due to an excessive immune response to external stimuli, can occur in both sexes (Demoly et al., 2014). Our study found that injection site erythema, bruising, pruritus, and mass can occur in both male and female; however, injection site rashes and urticaria were more common in women. The treatment of cutaneous hypersensitivity reactions depends on the severity, necessitating early intervention and management to prevent more serious systemic reactions. It is important to consider that the number and specificity of AE reports depend largely on individual reporting habits. We can only estimate the signal by its intensity and frequency. These results should not be overly relied upon to draw definitive conclusions about the causal relationship between drugs and adverse events. Therefore, it is not feasible to infer a causal relationship between medications and ADEs in male and female patients based solely on these data.

Our study analyzed the temporal relationship between drug administration and ADE onset time. The results showed that tirzepatide-related ADEs occurred predominantly within the first month (55.24%), and the Weibull distribution test indicated that the probability of ADEs decreased over time in the general population and in most subgroups. Tirzepatide is primarily used in patients with type 2 diabetes and obesity, necessitating long-term use, and these findings support the drug’s long-term safety. Crucially, we conducted a comprehensive study of the specific onset time in each organ system following tirzepatide administration. While most SOCs manifested within the first month of medication use, our results indicated that the median time to onset of hepatobiliary disorders was 35.5 days (IQR 4.75–154 days). This suggests the need for a follow-up program for early detection of hepatobiliary disease. Furthermore, SOCs with the longest time to onset were neoplasms benign, malignant, and unspecified (including cysts and polyps), suggesting that semiannual surveillance and follow-up may be warranted. Therefore, early detection and follow-up requirements for adverse reactions should vary depending on the affected systems. To investigate the effect of sex on the type of ADE, we performed a sex-based subgroup analysis, which suggested that the time to onset of ADEs associated with tirzepatide might not affected by sex. This underscores the importance of a uniform follow-up cycle for both men and women in monitoring ADEs.

While one study has already analyzed tirzepatide real-world data using the FAERS database (Liu, 2024), the significant increase in spontaneously reported ADEs for tirzepatide prompted us to comprehensively document and evaluate the drug’s safety for post-marketing use based on the largest sample of real-world data to date. However, several limitations remain. First, FAERS is inherently limited by underreporting, incomplete reporting, and selective reporting. Of the 37,827 reports we collected, the majority (93.3%) did not include outcome information, making more detailed analysis difficult. Second, the lack of detailed clinical information on patients, such as comorbidities, severity of underlying disease, and concomitant medications, limited our ability to control for confounding variables, which may have influenced the study results. Third, disproportionality analyses were limited to assessing signal strength and establishing statistical associations, which, despite our explanation in the Discussion section, did not provide sufficient evidence to establish a causal relationship between substance use and ADE. Lastly, the low reporting rate of TTOs (3.6%) represents a significant limitation of our analysis, and thus, these results require careful interpretation. Given these shortcomings, along with other potential confounders and biases, it is important to consider these limitations when interpreting our findings.

## Conclusion

Our study conducted a comprehensive and systematic pharmacovigilance analysis using the FAERS database to identify ADEs associated with tirzepatide. “injury, poisoning and procedural complications,” “general disorders and administration site conditions,” “gastrointestinal disorders,” and “metabolism and nutrition disorders” were the significant system organ classes with adverse events. Attention should also be directed towards unexpected signals, including “starvation ketoacidosis,” “medullary thyroid cancer,” and “follicular thyroid cancer”. We differentiated between sex-specific types of ADEs and examined the onset timing of these events. Given the inherent limitations of the FAERS database, potential confounders, and biases, our findings should be interpreted with caution. Further prospective clinical studies are necessary to confirm and enhance our understanding of the association between tirzepatide and these ADEs.

## Data Availability

The original contributions presented in the study are included in the article/Supplementary Material, further inquiries can be directed to the corresponding author.
